# Crosstalk of TGF-β and somatostatin signaling in adenocarcinoma and neuroendocrine tumors of the pancreas: a brief review

**DOI:** 10.3389/fendo.2025.1511348

**Published:** 2025-03-11

**Authors:** Hendrik Ungefroren, Harpal Randeva, Hendrik Lehnert, Jörg Schrader, Jens-Uwe Marquardt, Björn Konukiewitz, Ralf Hass

**Affiliations:** ^1^ Institute of Pathology, University Hospital Schleswig-Holstein (UKSH), Campus Kiel, Kiel, Germany; ^2^ First Department of Medicine, University Hospital Schleswig-Holstein (UKSH), Campus Lübeck, Lübeck, Germany; ^3^ University Hospital of Coventry and Warwickshire (UHCW) and Warwick Medical School, Coventry, United Kingdom; ^4^ First Department of Medicine, Universitätsklinikum Hamburg-Eppendorf (UKE), Hamburg, Germany; ^5^ Biochemistry and Tumor Biology Laboratory, Department of Obstetrics and Gynecology, Hannover Medical School, Hannover, Germany

**Keywords:** TGF-β, somatostatin, signaling, pancreas, pancreatic ductal adenocarcinoma, neuroendocrine tumors

## Abstract

Although the vast majority of cancers affecting the human pancreas are pancreatic ductal adenocarcinomas (PDAC), there are several other cancer types originating from non-exocrine cells of this organ, *i.e.*, pancreatic neuroendocrine tumors (panNET). Genomic analyses of PDAC and panNET revealed that certain signaling pathways such as those triggered by transforming growth factor-β (TGF-β) are frequently altered, highlighting their crucial role in pancreatic tumor development. In PDAC, TGF-β plays a dual role acting as a tumor suppressor in healthy tissue and early stages of tumor development but as a promoter of tumor progression in later stages. This peptide growth factor acts as a potent inducer of epithelial-to-mesenchymal transition (EMT), a developmental program that transforms otherwise stationary epithelial cells to invasive mesenchymal cells with enhanced metastatic potential. TGF-β signals through both the canonical Smad pathway involving the receptor-regulated Smad proteins, SMAD2 and SMAD3, and the common-mediator Smad, SMAD4, as well as Smad-independent pathways, *i.e.*, ERK1/2, PI3K/AKT, and somatostatin (SST). Accumulating evidence indicates an intimate crosstalk between TGF-β and SST signaling, not only in PDAC but, more recently, also in panNET. In this work, we review the available evidence on signaling interactions between both pathways, which we believe are of potential but as yet insufficiently appreciated importance for pancreatic cancer development and/or progression as well as novel therapeutic approaches.

## 1 Introduction

The vast majority of pancreas cancers (95%) are pancreatic ductal adenocarcinomas (PDAC). Thus, PDAC (and all of its clinical characteristics including its dismal prognosis) has become synonymous with “pancreatic cancer” (1–3). However, there are numerous other types of cancer originating from the pancreas, which are classified by their cellular lineage, i.e., acinar cell carcinomas (exhibiting acinar differentiation), neuroendocrine neoplasms (NEN, arising from islet cells) and - very rarely - pancreatoblastomas (showing multiphenotypic acinar, endocrine and ductal differentiation).

PDAC has an extremely poor prognosis - the 5-year survival remains below 10% - even when it is discovered in early stages. It is projected to become the 2nd leading cause of cancer deaths in the US by 2030 (2, 3). The treatment of PDAC remains one of the greatest challenges in oncology, since, despite the numerous advances made in pharmacology with the development of new drugs, this cancer remains refractory to oncological treatment. Surgical resection is the only curative option, however, only about 20% of patients are amenable to this treatment. While targeted therapies have successfully been introduced for cancers of breast, lung and colon, PDAC still relies predominantly on chemotherapy as standard care (1–3). Many trials have been carried out with novel drugs targeting metabolic pathways, epigenetic modifications, TP53 and claudins, but research in these fields is still in early stages. Combination of target therapies with chemotherapy and/or immunotherapy and more personalized approaches raise the hope to improve survival rates in PDAC.

**Figure 1:**
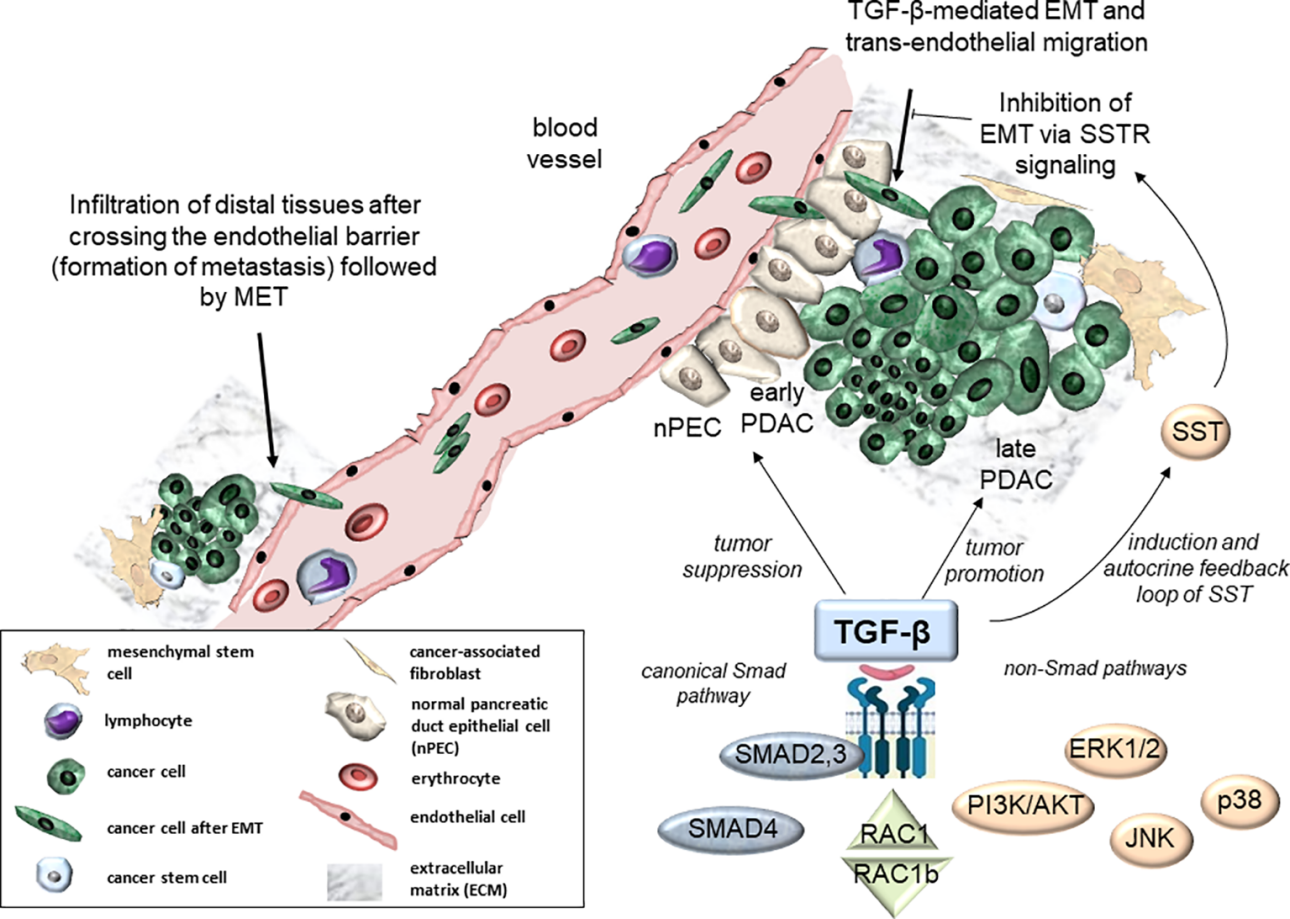
Molecular and cellular signaling effects of TGF-β in PDAC development. Molecular pathways of TGF-β include canonical Smad signaling (via SMAD2, SMAD3, and SMAD4) and non-Smad signaling via alternative routes such as PI3K/AKT, MAPKs (ERK1/2, p38, Jun N-terminal kinase (JNK)), SST, or RAC1/RAC1b (7–9) (bottom). At the cellular level, TGF-β is involved in tumor suppression of normal pancreatic tissue (nPECs) and early neoplasias (early PDAC, center). In contrast, TGF-β acts as a tumor promoter in advanced pancreatic tumors (late PDAC, right-hand side). Moreover, TGF-β contributes to trans-endothelial migration to support formation of distal metastases (top right). Conversely, SST can act via SSTR signaling to block TGF-β-mediated EMT-dependent processes (10) (top right). EMT, epithelial-mesenchymal transition; MET, mesenchymal-epithelial transition.

Pancreatic NEN (panNEN) make up 3–5% of all pancreatic cancers and represent a heterogeneous group of epithelial tumors with neuroendocrine differentiation. They are further classified into well-differentiated pancreatic neuroendocrine tumors (panNET), including G1, G2, and G3 tumors, and poorly-differentiated pancreatic neuroendocrine carcinomas (panNEC) (4, 5). PanNECs display histomolecular features more closely related to PDAC than to panNET, including TP53 and Rb alterations (4, 5) and it is thus plausible that PDAC and panNEC share in common the cells of origin. PDACs originate from ductal or acinar epithelial cells (6) (**Figure 1**) through a series of precursor lesions termed “pancreatic intraepithelial neoplasia” (PanIN) (2), while for panNET the cellular origin and the possible existence of precursor lesion(s) has remained unknown (5, 11). However, a recent study reports that panNET are derived from α cells, or immature or adult β cells of the islets (11).

PanNET are rare (incidence of <1 per 100,000 people) and can be classified into functional and non-functional subtypes according to whether or not the tumors secrete hormones (5). Functional panNETs account for 30%-40% of all panNETs (12) and comprise glucagonomas, insulinomas, gastrinomas, and somatostatinomas, named according to the hormone secreted. A recent molecular classification based on genomic, transcriptomic, proteomic and epigenomic studies of panNETs defined four subtypes with distinct molecular features (for details see (11)).

Interestingly, the very rare somatostatinomas secrete somatostatin (SST), which is a growth-hormone inhibitory peptide with antiproliferative properties. SST exerts direct anti-tumor effects by direct binding to and activation of one or more of five different SST receptors (SSTR1-5), with SSTR2 representing an inhibitory G protein-coupled receptor. In addition, SSTRs can also mediate indirect effects via growth factor regulation (13). The guidelines for the clinical management of panNET in the US recommend surgical resection for localized tumors and medical therapy with SST analogues (SSAs) for patients with functional tumors, Everolimus or Sunitinib for patients with unresectable or metastatic disease (14), and targeted therapy for patients with non-functional tumors or progressive disease (15). For most patients, only palliative treatments are available to successfully control the disease or to manage symptoms in functioning tumors. The synthetic SSAs octreotide (OCT) or lanreotide (LAN) are widely used and significantly improve the management of panNET. OCT, in addition, has been observed to inhibit tumor progression (see below).

Current knowledge about the molecular mechanisms driving PDAC and panNET generation and progression is still insufficient and novel therapeutic targets are urgently needed. Genomic analyses of PDAC revealed that signaling pathways of transforming growth factor (TGF)-β are altered in 100% of cases with at least one mutation (16, 17), highlighting the crucial role of this growth factor in PDAC development. TGF-β plays a dual role in PDAC, acting as a tumor suppressor in healthy tissue and in early stages of PDAC development but as a promoter of tumor progression in later stages (a phenomenon termed the TGF-β paradox). This paradox is also becoming obvious during TGF-β crosstalk with the small GTPase, RAC1, and its splice isoform, RAC1b, in PDAC (**Figure 1**) and mammary carcinomas (7) contributing to an antagonistic regulation of TGF-β-mediated growth arrest and migratory effects in normal and tumorigenic tissues (8, 9). Mechanistically, TGF-β signaling is a potent inducer of epithelial-to-mesenchymal transition (EMT), a developmental program that confers migratory and invasive properties to epithelial cells, hence aberrant TGF-β signaling and EMT are linked to promoting PDAC aggressiveness (**Figure 1**). TGF-β signals through both the canonical Smad pathway involving the receptor-regulated Smad proteins, SMAD2 and SMAD3, and the common-mediator Smad, SMAD4, as well as Smad-independent pathways, i.e., mitogen-activated protein kinases (MAPKs), MEK1/2-ERK1/2 and MKK3/6-p38, and PI3K/AKT and SST (**Figure 1**). Although each of these signaling pathways is by itself well characterized, their interplay during PDAC development and progression remains largely unclear. With respect to TGF-β and SST signaling, available evidence indicates an intimate crosstalk not only in PDAC but, more recently, also in panNET. Given the potential importance of signaling interactions between both growth factors in PDAC and panNET biology, we set out to compile the current state of knowledge on TGF-β/SST signaling interactions.

## 2 Expression of SST, TGF-β and their receptors in panNET and PDAC

Chaudhry and colleagues examined 23 midgut carcinoids and 7 panNETs for expression of TGF-β1, -2, -3 and TGF-β type II receptor (TβRII). Tumor cells from most tissues expressed all three isoforms of TGF-β. In stromal cells, abundant expression of TGF-β2 and TβRII was noted, whereas TGF-β1 and -3 were expressed only weakly. Tumor cells strongly expressed TGF-β2 and -3 but not TGF-β1, and were devoid of TβRII. Hence, TGF-βs might play an important role in the crosstalk of tumor and stromal cells by stimulating matrix production and angiogenesis in stromal cells (18).

To date, the cell line BON-1 has been the most widely used cellular model for panNET (19, 20). BON-1 cells and the primary human insulinoma cell line, NT-3 (21), produce and secrete SST and express a large variety of neuroendocrine markers, both constitutively and in response to treatment with OCT (21, 22). Like PDAC (see below), panNET can overexpress TGF-β and exhibit enhanced TGF-β signaling activity. For instance, serotonin producing tumors (SP-panNET) show TGF-β pathway activation signatures associated with extracellular matrix remodeling and desmoplasia (23).

Five SSTRs were cloned and termed SSTR1-SSTR5. SSTR1, SSTR2 and SSTR5 are thought to play major roles in inhibiting SST-induced PDAC growth both *in vitro* and *in vivo* (24). SSTR3 may be involved in mediating apoptosis, while the role of SSTR4 is unclear. In most PDACs, functional SSTRs are absent (25). Reintroduction of SSTR genes has been shown to inhibit PDAC growth in cell cultures and animal models (see below and (25)). Among the four human gastroenteropancreatic neuroendocrine tumors (gastrinomas, insulinomas, tumors with carcinoid syndrome, functionally inactive neuroendocrine tumors), expression levels of SSTR1, SSTR5, and TGFBR1 and TGFBR2 (encoding TGF-β type 1 receptor, also termed activin receptor-like kinase 5, ALK5, and TβRII, respectively) varied significantly, suggesting the existence of different pathways during tumor subtype development (26).

Human PDACs overexpress both TGF-β ligands and TβRII, which has been associated with decreased patient survival (27). TGF-βs bind to a TβRII dimer, which forms heterotetrameres with another dimer of TβRI/ALK5, thereby activating downstream signaling through Smad and/or non-Smad signaling intermediates.

Treatment with TGF-β1 of the TGF-β sensitive PDAC cell line, COLO 357 (harboring wild-type DPC4, the gene encoding SMAD4, and expressing relatively high basal levels of ALK5) caused a sustained increase in the mRNA and protein levels of both TβRII and ALK5 (28). From this observation, it was concluded that the TGF-β1-induced TβRII upregulation serves to enhance - via a transcriptional mechanism - TGF-β1 responsiveness in COLO 357 cells. This upregulation, which required the presence of adequate levels of ALK5 and TβRII besides a functional SMAD4 protein (29), has the potential to maximize TGF-β1-dependent cellular responses such as growth inhibition. In agreement with this assumption, upon orthotopic transplantation of individual PANC-1 clones stably expressing kinase-active ALK5 (ALK5T204D) into immunodeficient mice, this mutant, but not the Smad binding-defective derivative of ALK5T204D (RImL45T204D) greatly reduced tumor size but induced the formation of liver metastases in otherwise non-metastatic PANC-1 cells. These results suggest a causal, dominant role for the endogenous SMAD2/3 signaling pathway in the tumor suppressor and prometastatic activities of TGF-β in PDAC cells (30). Conversely, low levels of wild-type ALK5 (31) within PDAC tumors may protect against growth inhibition.

## 3 Effects of SST and SSTAs in tumor cells from the pancreas and pituitary, and crosstalk with TGF-β signaling

TGF-β regulates cell growth and differentiation in healthy tissues. In tumors of epithelial cell and neural cell origin, it still serves as a growth inhibitor in the early phases of tumor development but later becomes a growth promoter in transformed tumors. To accomplish this, the TGF-β receptors and the Smad proteins SMAD2/3/4 interact with other signal transduction cascades such as MEK1-ERK1/2, MKK3/6-p38, PI3K/AKT, RAC1 and Wnt/β-catenin just to name a few (32) (**Figure 1**). Alterations of the TGF-β/Smad signal transduction pathway, most notably loss-of-function mutations in, or genomic deletion of, DPC4 have been implicated in PDAC progression (32).

**Figure 2:**
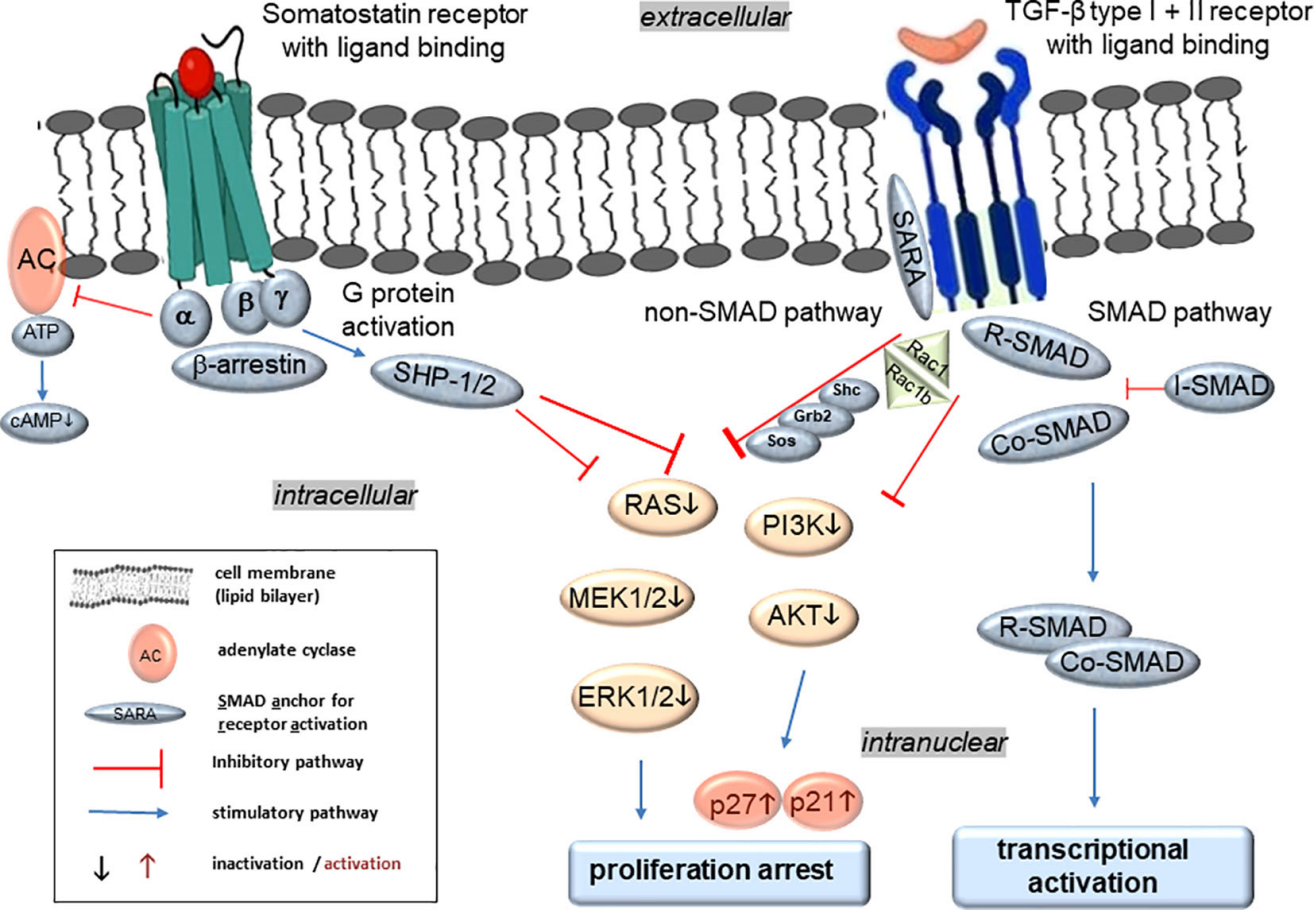
Molecular regulation of TGF-β and SST signaling in PDAC and panNET and hypothetical synergistic effects of their intracellular crosstalk. Activation of SSTRs after ligand binding leads to G protein activation that can subsequently inhibit adenylate cyclase (AC) and the formation of cyclic adenosine monophosphate (cAMP), which is required for protein kinase A (PKA) activity. Moreover, SSTR activation can increase tyrosine phosphatase activities (e.g., SHP-1/2), which in turn dephosphorylates and therefore inactivates downstream kinases such as MEK-ERK for the inactivation of protein and DNA synthesis. In addition, SHP-1/2-mediated dephosphorylation and inhibition of phosphatidylinositol-3-kinase (PI3K) and further AKT mediate enhanced expression of the cyclin-dependent kinase inhibitors p21^WAF1/CIP1^ (p21) and p27^KIP1^ (p27) with inhibition of proliferation. Ligand binding of the TGF-β receptors induces conformational changes to expose the kinase site of the cytoplasmic domain of ALK5 to appropriate substrate proteins for activation of a downstream signaling cascade via either the Smad or the non-Smad pathways. The non-Smad arm of TGF-β signaling can recruit various adaptor proteins such as Shc, Grb2, Sos, and RAC1 among others to relay downstream signals along branches of MAPK, Rho-like GTPase, and PI3K/AKT pathways. Like SSTR signaling, the TGF-β pathways can finally promote growth arrest. Alternatively, the Smad pathway of TGF-β signaling involves at least four different Smad proteins with three different functionalities: the receptor-regulated Smads (R-Smads) including SMAD2 and 3 are C-terminally phosphorylated by activated ALK5, subsequently form a complex with the Co-Smad, SMAD4, which is then translocated to the nucleus to modulate the activity of TGF-β target genes. In addition, the inhibitory Smad (I-Smad), SMAD7, lacks a C-terminal phosphorylation site and can function as a feedback inhibitor to terminate TGF-β receptor activation and hence signaling.

The binding of SSTRs to SST results in activation of various signaling pathways that regulate diverse cellular processes (**Figure 2**). The five receptors largely share in common the same signaling pathways, such as adenylate cyclase (inhibition), phosphotyrosine phosphatases, SHP-1 and 2 (activation), and MAPKs (activation or inhibition) through Gα/β/γ protein and β-arrestin-dependent mechanisms (33) (**Figure 2**). Specifically, SSTR5 activates the MEK-ERK, Jun N-terminal kinase (JNK) and p38 pathways to mediate proliferation arrest (**Figure 2**), and affects differentiation, apoptosis, anti-angiogenesis and tumor metabolism. All five SSTRs interfere with cell cycle progression by inhibition of MAPKs, PI3K/AKT and ultimately induction of the cyclin-dependent kinase inhibitors, p21WAF1/CIP1 and p27KIP1 (**Figure 2**), while SSTRs 2 and 3 promote apoptosis by activation of caspases (not shown). For instance, stable transfection of the PDAC cell line BxPC-3 with SSTR2 resulted in enhanced apoptosis induction by TNF-α, TRAIL or CD95L, and downregulation of VEGF and MMP-2 to reduce angiogenesis and metastasis (33). Last but not least, SSTR2 or 5 may adopt the role of an accessory receptor as previously shown for another G protein-coupled receptor, proteinase-activated receptor 2 (PAR2). PAR2 was identified as a factor required for TGF-β1-dependent cell motility in PDAC cells through its ability to positively control the expression of ALK5 (34).

Much like TGF-β, SST exhibits both paracrine and autocrine modes of signaling, which in normal and precancerous cells display well-established antiproliferative effects. However, SST signaling is also involved in mediating other inhibitory effects on cancers. In PDAC, it interferes with the progression of the EMT program (**Figure 1**), or even reversed TGF-β-induced EMT (a process named mesenchymal-epithelial transition - MET) in the cancer cells. The complex interplay between EMT, genetic and epigenetic alterations, and various signals from the tumor microenvironment that also mediate MET, further shape cancer cell plasticity (35). Interactions of cancer cells with other cellular partners in the tumor microenvironment, predominantly mesenchymal stem cells (32), further contribute to tumor heterogeneity and enhanced cancer cell plasticity (36, 37), while mesenchymal stem cell-derived exosomes provide therapeutic vehicles (38).

In PDAC cells, SST downregulates TGF-β/Smad pathway activation by reducing the abundance of the TGF-β1 ligand and/or SMAD2/3 (10). Moreover, SST suppresses their growth and metastatic capacity, and enhances the pro-apoptotic effects of TGF-β by upregulating the expression of the pro-apoptotic proteins caspase-3 and BAX, and downregulating that of the anti-apoptotic protein BCL-2. Finally, certain transcription factors known to be activated by TGF-β are also targeted by individual SSTRs, i.e., SP1 by SSTR1/3/5, p53 by SSTRs 4 and 5, and SMAD5 by SSTR3 (11).

Leu and colleagues explored the interplay between the SST and TGF-β signaling pathways in panNET using BON-1 cells (39). SST signaling was shown to be crucial for the cells’ growth-inhibitory response to TGF-β. In turn, TGF-β induces the production of SST and potentially activates the negative autocrine loop of SST (**Figure 1**), which led to the downstream induction of cell cycle inhibitors, p21WAF1/CIP1 and p27KIP1 (**Figure 2**), and downregulation of the growth accelerator, c-MYC, together establishing a cytostatic effect on BON-1 cells (39). Any disruption in the activation of either the TGF-β or SST signaling pathway resulted in a “reversible” neuroendocine-mesenchymal transition, which is characterized by the loss of neuroendocrine markers and an increase in the expression of mesenchymal markers (i.e., vimentin and Twist) and a decrease in the expression of epithelial markers such as E-cadherin (39). Since E-cadherin is considered an invasion suppressor, its downregulation has been associated with elevated metastatic potential. Hence, TGF-β-dependent growth inhibition and differentiation is mediated by the SST signaling pathway and any disruption of this TGF-β-SST crosstalk allows BON-1 cells to respond to TGF-β as a growth promoter rather than a growth suppressor (39). This model may also apply to primary panNET cell types, i.e., NT-3 (20–22).

The molecular mechanisms of the SSA and OCT leading to successful disease control or symptom management are largely unknown. This applies, in particular, to conditions where SSTRs levels are low. Although not of pancreatic origin, interesting observations were made in the midgut carcinoid cell line, CNDT2.5 (histological origin questionable). Midgut carcinoids originate in the small intestine and are the most common cause of the carcinoid syndrome (40). CNDT2.5 cells were treated for up to 16 months with OCT and profiled with Affymetrix microarray analysis. Although this approach failed to reveal any relevant changes in SSTR expression levels, it unexpectedly identified six novel genes found to be upregulated by OCT. Of note, these genes included two members of the TGF-β family of ligands, namely GDF15 (encoding Growth Differentiation Factor 15) and TGFBR2 (encoding TβRII). To regulate cell growth and differentiation in normal and neuroendocrine tumor cells, OCT may thus use a novel framework to exert its beneficial effect as a drug (41). Since even cells with low-level expression of SSTRs may exhibit significant responses to OCT, it is likely that SSAs signal through alternative mechanisms, e.g., TGF-β. In the meanwhile, the involvement of TGF-β in SSTR signaling has been demonstrated in BON-1 and NT-3 cells (22, 39).

We also observed that SST, OCT and LAN regulate a set of neuroendocrine genes in both BON-1 and NT-3 cells. However, unlike NT-3, BON-1 cells failed to respond to OCT with growth arrest, while LAN even exhibited a growth-stimulatory effect. Following treatment with TGF-β1, BON-1 and NT-3 cells reacted with induction of SST and SSTR2/5, and upregulation of SERPINE1 (encoding plasminogen activator-inhibitor type 1, PAI-1), the latter effect of which depended on cellular adherence to a collagen-coated matrix (22). Moreover, when applied to NT-3 cells for a period of 14 days, TGF-β1 induced growth suppression (20) as shown earlier for BON-1 cells (39). NT-3 cells also responded in a similar fashion as BON-1 cells to treatment with SST, SSA, or TGF-β1. Hence, crosstalk of SST and TGF-β signaling appears to be a general feature of panNET (22).

OCT is also used in somatotroph tumors to inhibit hormone secretion and growth, although a significant percentage of patients are resistant to this treatment. This SSA has also been tested in non-functional tumors but with poor results, which was probably caused by low SSTR2 levels and/or impaired signaling. A recent study, therefore, investigated whether OCT inhibitory effects can be improved by co-administration of TGF-β1 in functional and non-functional somatotroph tumor cells (42). To this end, the effects of OCT on hormone secretion and proliferation were analyzed in the presence of TGF-β1 in both wild-type and SSTR2 overexpressing secreting and silent GH3 cells, a lacto-somatotroph tumor cell line from the rat pituitary gland. The mechanism underlying these effects was assessed by studying SSTR and TGF-β signaling mediators. In addition, the effects of combined OCT/TGF-β1 treatment on tumor growth and cell proliferation *in vivo* were analyzed (42). Of note, the inhibitory effects of OCT on growth hormone and prolactin secretion and proliferation of GH3 cells were relieved by the addition of TGF-β1 or by ectopic overexpression of Sstr2 (42). The combined OCT/TGF-β1 treatment induced downregulation of phospho-Erk1/2 and phospho-Akt, upregulation of phospho-Smad3, and inhibition of cyclin D1 (42). *In vivo* experiments showed that OCT+TGF-β1 blocked an increase in tumor volume by decreasing cell proliferation and enhancing tumor necrosis (42). Hence, levels of Sstr2 and activation of the TGF-β1/TβR/Smad2/3 pathway are important for strengthening the anti-proliferative and anti-secretory effects of OCT (42).

## 4 SSTR2: Regulation by TGF-β

SSTR2 has been found to exhibit anti-tumor properties, however, its expression is lost in most human PDACs (43), suggesting transcriptional silencing as the underlying mechanism. To test this possibility, Puente and colleagues set out to clone approx. 2 kbp of mouse genomic DNA upstream of the Sstr2 translation initiation site. Deletion reporter analyses in AtT-20 murine pituitary cells and in the human PDAC cell lines, PANC-1, BxPC-3 and CAPAN-1, identified a region extending from nt -260 to the ATG start codon with maximal transcriptional activity, and a region between nts -2025 and -260 that may contain silencer elements. Interestingly, in PANC-1 and AtT-20 cells, treatment with TGF-β1 upregulated murine Sstr2 transcription. In agreement with responsiveness to TGF-β1, the Smad4 and Smad3 proteins were able to transactivate Sstr2, which involved a cis-acting region between nts -1115 and -972 containing binding sites for the transcription factor Sp1 as well as CAGA-box sequences. Notably, ectopic re-expression of SMAD4 in DPC4-deficient CAPAN-1 and BxPC-3 cells restored TGF-β1-dependent and -independent SSTR2 transactivation. Moreover, forced expression of SMAD4 in BxPC-3 cells re-established both endogenous SSTR2 expression and SST-mediated inhibition of cell growth. Hence, murine Sstr2 appears to be a novel target gene for transcriptional regulation by TGF-β1 and suggests that loss of DPC4/SMAD4 contributes to the lack of SSTR2 expression in human PDAC. This loss may, in turn, contribute to enhanced tumor growth due to the inability of TGF-β1 to exert growth inhibitory effects (43).

## 5 SSTR2, TβRII and ALK5: Antitumor effects

SST and TGF-β1 mediate their effects through high-affinity receptors. Among the five SSTRs, the SSTR subtype 2 was found to be expressed at relatively high levels in pancreatic tumor cells and tumor blood vessels (44) and the loss of SSTR2 promoted the development of human PDAC (45) (see 5.2.). TGF-β1 mediates its cellular effects via two membrane-anchored serine/threonine kinase receptors, TβRII and ALK5, that collaborate to trigger signaling via Smad proteins or a variety of non-Smad pathways (**Figure 1**). It is interesting to note that 24/24 (=100%) of analysed advanced PDACs contained genetic alterations in at least one of all genes involved in TGF-β signaling, highlighting the crucial role of this pathway, and hence TβRII and ALK5, in this tumor type (16).

There is also evidence for signaling crosstalk between SSTR2 and the TGF-β receptors (**Figure 2**). BON-1 and NT-3 cells responded to TGF-β1 stimulation with production of SST and SSTR2/5 (22, 39), and TGF-β1-dependent growth arrest and differentiation was mediated by SST signaling (likely acting via SSTR2) (39). Moreover, in PDAC cells treated with SST, the TGF-β1, SMAD2 and SMAD3 proteins were downregulated (10). Finally, murine Sstr2 has been identified as a target gene of transcriptional regulation by TGF-β (43). Very recently, we have shown in PANC-1 cells that TGF-β1 upregulates SSTR2 and downregulates SSTR5 at the mRNA level (46).

### 5.1 In cancer cells *in vitro*

#### 5.1.1 SST/SSTR2

As a consequence of losing the ability to express SSTR2 human PDACs were shown to be unable to respond to SST’s antiproliferative effect (43). Stable expression of SSTR2 in human PDAC cells, or Sstr2 in hamster PC-1 and PC-1.0 pancreatic cancer cells, led to constitutive activation of SSTR2/Sstr2 and activation of a stable autocrine negative loop that restored the growth inhibitory effect and decreased tumorigenicity. As mentioned above, ectopic SSTR2 can also mediate the anti-proliferative effect of SSAs and strongly reduce the invasive activity of PC-1.0 cells *in vitro*. The latter response may be due to restoration of E-cadherin function via SHP-1-dependent tyrosine de-phosphorylation of E-cadherin (47).

Reintroduction and expression of human SSTR2 in BxPC-3 cells had anti-migratory and anti-invasive effects through downregulation of MMP2 (encoding matrix metallopeptidase 2; MMP-2), and upregulation of TIMP2 (encoding tissue inhibitor of metalloproteinase 2, TIMP-2) (48), two matrix-associated proteins that are also (positively) targeted by TGF-β. OCT treatment of these SSTR2 transfected cells significantly decreased the IC50 of chemotherapeutic agents, cisplatin, epirubicin, 5-fluorouracil, and gemcitabine in SSTR2-expressing BxPC-3 cells in a dose-dependent manner. Mechanistically, a decrease in the expressions of different multidrug resitance (MDR) genes, namely multidrug resistance gene-1 (MDR1), multidrug resistance-associated protein 2 (MRP2) and lung resistance related protein (LRP) following human SSTR2 transfection, and an enhancement of this effect by an 48-hour treatment with OCT, were identified as being responsible for the increased chemosensitivity of SSTR2 transfected BxPC-3 cells (49).

#### 5.1.2 TβRII+ALK5

Clones of the PDAC-derived cell line, COLO 357, expressing a soluble form of TβRII (sTβRII, encoding amino acids 1-159 of the extracellular domain) that blocks cellular responsiveness to TGF-β1, were no longer growth-inhibited by exogenous TGF-β1 and showed a marked decrease in their invasive capacity *in vitro*. Ectopic expression of dominant-negative mutants of TβRII (D404G mutation) or kinase-dead ALK5 (K232R mutation) in TGF-β sensitive PANC-1 cells prevented the TGF-β1-induced activation of transfected Smad-responsive reporter genes and growth arrest (30).

The growth-inhibitory effect of TGF-β1 via wild-type TβRII in PANC-1 cells was mimicked by stable ectopic expression of kinase-active ALK5 (ALK5T204D). To explore if this effect was dependent on ALK5’s ability to activate Smad signaling, we stably expressed a mutant form of ALK5 with an intact and active kinase domain but deficient in its ability to activate SMAD2 and SMAD3 due to a mutation in the L45 loop (RImL45-T204D) (50). This selective interference with endogenous SMAD2/3 activation rendered ALK5T204D unable to suppress proliferation in the absence of exogenous TGF-β1 (30). Moreover, ALK5RImL45/T204D often displayed opposite effects to those of ALK5T204D and blocked various ligand-induced responses *in vitro*, indicating that it acts in a dominant-negative fashion to inhibit endogenous wild-type receptors. ALK5T204D but not ALK5RImL45/T204D-transduced cells underwent EMT, presented with a higher ratio of thrombospondin-1 (TSP1) to VEGF-A expression and upregulated various metastasis associated genes (30). This suggested that SMAD2 and/or SMAD3, but not necessarily SMAD4, function (51) is required for EMT induction.

SD-208, a 2,4-disubstituted pteridine, ATP-competitive inhibitor of the ALK5 kinase, was used to inhibit cellular activities and tumor progression of PANC-1 cells. This drug blocked TGF-β-dependent SMAD2 C-terminal phosphorylation and expression of TGF-β-inducible proteins in cell culture. A cDNA microarray analysis and functional gene clustering identified a series of TGF-β-regulated genes involved in controlling neo-angiogenesis, cell proliferation and survival, and metastasis. As these genes were inhibited by SD-208, it was not surprising that SD-208 also inhibited TGF-β1-stimulated invasion *in vitro* (52).

### 5.2 In experimental tumor models *in vivo*

#### 5.2.1 SST/SSTR

*In vivo* SSTR2 transfer into human PDAC tumors strongly inhibited tumor growth and progression by inducing intra-tumoral production of SST. Disruption of this autocrine loop by RNA interference completely reversed SSTR2 anti-tumoral activity (53). To study the anti-oncogenic effects of Sstr2 in primary tumors in more detail, two PDAC models established in nude mice and hamsters were employed. Expression of cloned Sstr2 induced both anti-oncogenic and local anti-tumor bystander effects *in vivo* (see also section 5.3). *In vivo* gene transfer of Sstr2 was also investigated in two transplantable hamster models, one for primary and one for metastatic PDAC (54). Murine Sstr2, or LacZ reporter as control, was expressed by means of two different delivery agents, an adenoviral vector and a synthetic polycationic carrier (PEI). Sstr2 was injected into either exponentially growing pancreatic primary tumors or hepatic metastases followed by investigation of transgene expression and tumor progression 5-6 days post-injection. Both adenoviral vector and PEI-based Sstr2 transfer resulted in growth reduction of pancreatic primary and metastatic tumors (54). In tumors ectopically expressing Sstr2, the proliferative index decreased, while the apoptotic one increased, and was associated with an activation of the caspase-3 and poly(ADP-ribose)-polymerase (PARP) pathways. Hence, in both primary and metastatic PDAC models, *in vivo* Sstr2 transfer induced a significant anti-tumor effect that resulted from a decrease in cell proliferation and an increase in apoptosis (54).

As mentioned above, expression of SSTR2/Sstr2 is progressively lost during human PDAC development. In mice with an oncogenic Kras (G12D mutation) genetic background, Sstr2 loss led to an increase in the activity of Pi3k and progression of premalignant to neoplastic lesions and PDAC (45). Using mice expressing KrasG12D in pancreatic precursor cells (KC (55)), mice with monoallelic loss of Sstr2 (Sstr2+/–), and crossed KrasG12D/Sstr2+/- mice, this group in more mechanistic detail analysed the effects of Sstr2 loss on tumor growth in the course of Kras-induced PDAC development. In crossed KrasG12D/Sstr2+/- mice, activation of Pi3k/Akt signaling was enhanced and premalignant lesions, tumors, and lymph node metastases developed more rapidly than in KrasG12D mice. This was due to subsquent activation of nuclear factor (NF)-κB, which then increased KRAS activity and its downstream pathways, which ultimately promoted the initiation and the transformation of premalignant to neoplastic lesions. This activation loop was mediated by Pi3k-induced synthesis of the chemokine Cxcl16. Hence, loss of Sstr2 from murine pancreatic tissues resulted in the following sequence of signaling activation that culminated in increased expression of Cxcl16 and pancreatic tumor formation: Pi3k/Akt, NF-κB, oncogenic Kras (45). Since activation of Pi3k is required for Kras induction and maintenance of PDAC in mice but is inhibited by Sstr2, these findings offer a mechanistic explanation of why expression of oncogenic Kras in the presence of ongoing Sstr2 expression is sufficient to initiate carcinogenesis but not progression to cancer.

#### 5.2.2 TβRII

Mice with a homozygous deletion of Tgfbr2 combined with activated Kras (KrasG12D) expression developed well-differentiated PDAC with 100% penetrance. Heterozygous deletion of Tgfbr2 with KrasG12D expression also induced PDAC, which indicated a haploinsufficiency of TGF-β signaling in this genetic context. The clinical and histopathological manifestations of the combined KrasG12D expression/Tgfbr2 knockout in mice thus recapitulated human PDAC carcinogenesis. Hence, blockade of TGF-β signaling and activated Ras signaling cooperate to promote PDAC progression (56).

When sTβRII expressing clones of COLO 357 cells were injected subcutaneously into athymic mice, they exhibited attenuated growth rates and angiogenic activities (57). This was accompanied by decreased mRNA levels of the TGF-β response gene SERPINE1, which encodes PAI-1, a marker of angiogenesis and metastatic capacity (57). Likewise, when sTβRII-expressing clones of another PDAC line, PANC-1, were studied in an orthotopic mouse model, they formed smaller intrapancreatic tumors than controls and their metastatic capacity and expression of the metastasis associated SERPINE1 and PLAU genes were suppressed. Together, these results indicate that *in vivo*, endogenous TGF-βs can confer a growth advantage to PDAC cells that are growth inhibited *in vitro* and suggest that blocking TβRII function can be employed to inhibit these tumorigenic effects of TGF-βs (57).

#### 5.2.3 ALK5

In previous work we studied the effects of the TGF-β type I receptor, ALK5, on PDAC tumor cell growth and metastasis. Upon orthotopic transplantation of PANC-1 clones stably expressing a kinase-active ALK5 mutant (ALK5T204D) into immunodeficient mice, these clones grew to tumors of greatly reduced size and formed liver metastases in otherwise poorly metastatic PANC-1 cells (30). Previously, it was demonstrated in breast cancer (BC) cell lines grown as xenografts that SMAD2/3 signaling played a dominant role in mediating tumor suppressor effects on well-differentiated BC lines but pro-metastatic functions on a more invasive, metastatic BC cell line (50). Specifically, reduction in SMAD2/3 signaling by ectopic expression of ALK5RImL45/T204D enhanced the malignancy of xenografted tumors of the well-differentiated MCF10A-derived BC cell line, MCF10CA1h, resulting in formation of larger tumors with a higher proliferative index and more malignant histologic features (50). In contrast, expression of ALK5RImL45/T204D in the more aggressive MCF10CA1a cell line strongly suppressed formation of lung metastases following tail vein injection (50).

Since PANC-1 cells are representative of the quasi-mesenchymal subtype with a complete EMT and a metastatic phenotype (58), we asked whether in PDAC, too, ALK5-dependent SMAD2/3 signaling is required for ALK5 to mediate pro-metastatic effects. To this end, in PANC-1 clones orthotopically transplantated into SCID/Beige mice, ALK5T204D but not ALK5RImL45/T204D, greatly reduced tumor size and induced the formation of liver metastases (30). These results suggest a causal, dominant role for the endogenous SMAD2/3 signaling pathway in the tumor suppressor and pro-metastatic activities of TGF-β in PDAC cells.

Moreover, a murine orthotopic xenograft model of PDAC with PANC-1 cells revealed that the pharmacological ALK5 kinase inhibitor, SD-208 (59), reduced primary tumor weight and decreased the incidence of metastasis. This demonstrates the crucial role of TGF-β/ALK5 signaling in promoting tumor progression in established PDACs (52). However, both, ectopic expression of ALK5T204D or treatment with SD-208 reduced the size/weight of the primary tumor, which is an obvious contradiction. Since both the cell line (PANC-1) and the *in vivo* model (orthotopic xenotransplantation in mice) were identical, an explanation for this discrepancy is not immediately apparent but might be explained by the observation that the anti-tumor activity of SD-208 is dependent on the microenvironment (60).

#### 5.2.4 SST

Unfortunately, SST signaling was not analyzed in any of these studies thus not allowing conclusions as to whether SSTR2 signaling crosstalks with TβRII or ALK5 in tumor suppression or with ALK5 in metastasis promotion. This certainly is an area for future research.

### 5.3 Bystander effects

Bystander effects are biological responses in cells that are not themselves manipulated, e.g., by ectopic expression of a foreign gene, but receive signals transmitted from the manipulated (e.g., transfected) cells. This phenomenon has been intensively studied in radiotherapy of cancer. Irradiation-induced non-targeted/bystander effects (IRIBE) occur in a cell that is not directly traversed/hit by ionizing radiation (IR), but resides in the vicinity of one that is, or that has received signals from such cell(s), and can participate in the damage response. These bystander cells will amplify or exaggerate the responses of the transfected (or irradiated) cells and thus can significantly increase the cellular or tissue response (or radiation risk and tissue damage in case of a radiotherapy (61)). Data on IRIBE are only available for PDAC but not other pancreatic cancer types.

#### 5.3.1 SST

*In vivo* studies have shown that ectopic *SSTR2* expression in PDAC cells, e.g., human BxPC-3 or hamster PC-1.0 cells, can induce anti-tumor bystander effects. Specifically, after orthotopic implantation of PC-1.0 cells into Syrian golden hamsters both tumor growth and metastatic progression of allografts containing 100% of *SSTR2* expressing cells were inhibited for up to 20 days after implantation. A *local* anti-tumor bystander effect was also observed after induction of mixed tumors containing an only 1:3 ratio of *SSTR2* expressing to control cells in that tumor volume and incidence of metastases were significantly reduced already at day 13 post-implantation. An increase and decrease in the apoptotic and proliferative activity, respectively, was also noted in mixed and *Sstr2*-only tumors when compared to control tumors. In mice separately xenografted with control cells on one flank and *SSTR2* expressing cells on the other flank, an anti-tumor effect was induced: the growth of control tumors (in animals that also received *SSTR2* expressing cells) was delayed by 33 days and was associated with decreased and increased indices of proliferation and apoptosis, respectively, when compared with control tumors that grew alone. This so-called *distant* bystander effect may be explained in part by elevated serum levels of an SST-like immunoreactivity. These, in turn, may have arisen by the autocrine feedback loop generated by *SSTR2* expressing cells with upregulation of *SSTR1*, since SSTR1 can also mediate anti-proliferative effects of SST (62). Interestingly, after administration of the cytotoxic SST conjugate, AN-238 (doxorubicin-SST conjugate synthesized by coupling pyrrolino-doxorubicin to the SST analog RC-121), on day 13, the anti-tumor bystander effect observed in mixed tumors was significantly extended to day 20. Anti-tumoral bystander mechanisms also suppressed tumor angiogenesis in peripheral tumor vessels; *in vivo SSTR2* transfer into human PDAC tumors markedly reduced microvessel density and VEGF expression, while *Sstr3* was upregulated. Hence, VEGF, tumor vascularization, and Sstr3 expression were identified as novel targets for the *SSTR2-*mediated anti-tumor bystander effect (53). Taken together, the inhibitory effect of SSTR2 gene expression on PDAC growth, invasion, and neo-angiogenesis combined with chemotherapy using targeted cytotoxic SST administration provides a rationale for a therapeutic approach to *in vivo SSTR2* transfer for PDAC patients with unresectable disease (47, 53).

#### 5.3.2 TGF-β

TGF-β1 plays crucial roles in mediating IRIBE, which often involve negative or positive feedback loops as seen with SST signaling (see above). Although not performed in animal models of PDAC, studies in those of other cancer types have shown that TGF-β1 is induced by radiotherapy within the directly irradiated cells. Here, TGF-β signaling mediates IRIBE (63–65), which can promote tumor metastasis contributing to the failure of radiotherapy for esophageal squamous cell carcinoma (ESCC). Mechanistically, IR can induce ESCC cells to secrete DJ-1, which causes bystander cells to initiate activation of the TGF-β1 pathway via the DJ-1/HSC70/SMAD3 signaling axis. SMAD3 activation by C-terminal phosphorylation then activates transcriptional activity of *THBS1* (encoding TSP1). Subsequently, the proteolytic activation of latent TGF-β1 by TSP1 (66) re-promoted SMAD3 activation and nuclear translocation, constituting a positive feedback loop to strengthen the metastasis of ESCC cells (63).

#### 5.3.3 MicroRNAs

microRNA (miR)-663a is a radiosensitive miR that participates in the regulation of biological effects in both directly irradiated and bystander cells by targeting TGFB1. In directly irradiated cells, miR-663a was downregulated, while TGFB1 was upregulated. In contrast, in bystander cells this mode of regulation was reversed, with elevation of miR-663a expression leading to suppression of TGFB1 via direct binding of miR-663a to the core regulation sequence of TGFB1. Hence, miR-663a inhibits the propagation of IRIBE in a feedback mode, in which the induction of TGFB1 by reduced levels of miR-663a in directly irradiated cells led to increased levels of miR-663a in bystander cells. Conversely, upregulation of miR-663a suppressed the expression of TGFB1 and limited further transmission of the bystander signals (64). Of note, silencing of TGFB1 by miR-663a reversed the EMT triggered by IR and blocked the associated increase in cell migration (65).

Other IRIBEs involving TGF-β signaling include miR-21 (67, 68), reactive oxygen species (ROS) (67), nitric oxide (NO)/cGMP (69), cyclooxygenase 2 (COX-2) (70) or clusterin (71).

## 6 Conclusions and perspectives

Numerous trials with novel targeted drugs have been carried out aiming at improving overall survival and response rates in PDAC patients. The genetic landscape of PDAC has been widely analyzed leading to the identification of frequently mutated genes, including KRAS, TP53, CDKN2A, and DPC4. In addition, there are other more rarely mutated genes, which are promising therapeutic targets, such as BRAF, FGFR1, MYC, MDM2, BRCA1/2, ATM and mismatch repair genes (3). Novel targets that are currently being explored in PDAC include PARP, EGFR, HER2, claudin as well as regulators of (neo-) angiogenesis, metabolism, epigenetics, and their respective downstream and upstream pathways. However, SST, SSAs or SSTRs are not currently being evaluated in experimental models or clinical trials of PDAC (3). Conversely, TGF-β inhibitors for cancer therapy have been considered by pharmaceutical companies, and some of them are still being investigated in clinical trials, although not all drugs have met the high expectations and, as a consequence, progress in this field has slowed down (72).

TGF-β favors an immunosuppressive microenvironment and is an established promoter of immune evasion in PDAC. Inhibiting TGF-β has been shown to augment therapeutic responses to immune checkpoint inhibitors (ICIs) in preclinical models of disease and combined TGF-β and PD-1/PD-L1 inhibition is now emerging in clinical trials for PDAC (73). In this context it is interesting to note that SSTR2 expression has recently been identified as a potential predictive biomarker for ICI treatment response (74). However, the addition of TGF-β inhibitors has often failed to show a clinically relevant benefit (beyond that of the current generation of ICIs alone) due to lack of efficacy or poor activity, or patients even experienced unwanted side effects when physiological benign functions of TGF-β were compromised. Likewise, the combination of TGF-β inhibitors and SST/SSAs could potentially increase the risk of harmful side effects, such as inflammation or autoimmunity.

The use of TGF-β inhibitors in immune-oncology is primarily intended to relieve immunosuppression and restore anti-cancer immune responses, e.g., by restoring activation of CD4+, and CD8+ cytotoxic T cells reactive to tumor antigens, and decreasing the generation of regulatory T cells. However, this therapeutic goal of anti-TGF-β drugs might by compromised upon co-application of SSAs, since OCT has been found to exert antiproliferative effects on human lymphocytes and to enhance IL10 (which is besides TGF-β one of the most potent anti-inflammatory cytokines), and to inhibit pro-inflammatory IFNγ (75). Of note, the growth-inhibitory action of OCT on T cells is caused by induction of apoptosis and mediated by the SSTR2 subtype, SSTR2a (75). Biochemically, the suppressive influence of SST on T cell responses and metabolism is associated with a reduction in mitochondrial respiration through an SSTR3-induced activation of glycogen synthase-kinase 3 (GSK3) (76).

The EMT program is associated with an immunosuppressive microenvironment and promotes cancer progression by inhibiting multiple apoptotic signaling pathways and enhancing drug efflux and cancer stem cell generation. All these mechanisms contribute to the cancer cells’ increased resistance to anti-cancer drugs. In addition, EMT upregulates several pathways that allow cancer cells to resist the lethal effects of cytotoxic T cells, thus enhancing resistance to immunotherapy with different ICIs, including PD-L1. Much like SSTR2 expression in immunotherapy, an EMT-related gene signature predicts response to adjuvant chemotherapy (77) and blockage of EMT by the highly-specific doublecortin-like kinase 1 (DCLK1) inhibitor (DCLK1-IN-1) restored T cell activity and the response to ICIs (78).

Since both anti-TGF-β agents and SST/SSAs (10) inhibit the EMT program of pancreatic cancer cells, combined treatment should mutually enhance their beneficial therapeutic effects on the immune system. Apart from the antiproliferative effects of OCT on human T cells (75), neither general immunosuppression nor other major side effects have been associated with SST/SSA therapy. Moreover, in non-cancer pathologies, such as liver fibrosis, both SST/SSAs/OCT (79) and TGF-β inhibitors (80) display beneficial therapeutic effects. However, since combined therapies have not yet been tested clinically in greater depth, we must certainly advise to be aware of their potential adverse effects on TGF-β’s normal physiologic/homeostatic functions in various tissues and multiple processes, including its tumor suppressor effect.

These drawbacks also underscore the need for re-evaluating the design of trials exploring this approach, incorporating both mechanism-driven combination strategies and novel, predictive biomarkers to identify the patients most likely to benefit.

In the light of TGF-β being a pleiotropic cytokine with often contradictory or context-specific roles in tumorigenesis, one may argue that a more thorough understanding of its biology, including its multiple interactions with other signaling pathways, must be achieved before its full potential in combination therapies can be exploited. In this context, SST-TGF-β signaling crosstalk has remained understudied. Intriguingly, virtually all cellular responses to SST/SSA treatment or SSTR2 transfection observed in cancer cells *in vitro* or in animal models of cancer *in vivo* (i.e., tumor growth/weight, cell proliferation, apoptosis, migration/invasion/metastasis, angiogenesis, expression of SST/SSTR or TGF-β/TβRs) are also regulated by TGF-β. Not surprisingly, SST and TGF-β signaling target the same specific genes and proteins (i.e., PAI-1, uPA, VEGF, TGF-β1), signaling mediators (e.g., ROS (81), NO/cGMP (82), COX-2 (83–85)), and utilize the same molecular mechanisms (i.e., transcriptional induction, bystander effects). Therefore, both signaling pathways may have a degree of crosstalk that is not yet appreciated, a hypothesis that will hopefully be addressed in the near future. It is also conceivable that TGF-β affects the outcomes of systemic SSA treatments in panNETs, probably hindering an increase in overall survival and/or therapeutic efficacy. Clearly, much more research is required to dissect the signaling crosstalk mechanisms to evaluate whether these can be exploited to the benefit of PDAC and panNET patients.
